# ﻿A revision of some species of *Souvanna* Breuning, 1963, *Mispila* Pascoe, 1864, and *Athylia* Pascoe, 1864 (Coleoptera, Cerambycidae, Lamiinae)

**DOI:** 10.3897/zookeys.1190.115573

**Published:** 2024-01-24

**Authors:** Gui-Qiang Huang, Andreas Weigel, En-Ming Chang, Gui-Mei Zhang

**Affiliations:** 1 School of Biological Science and Technology, Liupanshui Normal University, Liupanshui 553004, Guizhou, China Liupanshui Normal University Liupanshui China; 2 Am Schloßgarten 6, D-07381 Wernburg, Germany Unaffiliated Wernburg Germany

**Keywords:** Gender definition, lectotype designation, new combination, new faunistic records, synonyms

## Abstract

*Alidussignatus* Pic, 1926 is transferred from *Mispila* to *Souvanna*, and *Souvannasignata* (Pic, 1926), **comb. nov.** is proposed. The lectotype of *Alidussignatus* is designated. The following synonyms are proposed: *Souvannasignata* = *Athylia* (*s. str.*) *quadristigma* (Gressitt, 1940), **syn. nov.** = *Souvannaphoumai* Breuning, 1963, **syn. nov.** = Mispila (Dryusa) coomani Breuning, 1968, **syn. nov.**, *Mispila* (*s. str.*) *tenuevittata* (Pic, 1930) = *Mispila* (*s. str.*) *assamensis* Breuning, 1938, **syn. nov.** The gender of the holotype of *Alidusmultilineatus* Pic, 1925 is determined. New distributional records for *Souvannasignata*, *Mispilacurvilinea* Pascoe, 1869, *M.subtonkinea* Breuning, 1968 and *M.tenuevittata* are provided.

## ﻿Introduction

Breuning (1963a) established the monotypic genus *Souvanna* for *Souvannaphoumai* from Laos (Vientiane). Pascoe (1864) established the genus *Athylia* for *Athyliaavara* Pascoe, 1864. Gressitt (1940) described *Enispiaquadristigma* from China (Hainan), and Breuning (1960) transferred it to the genus *Athylia* Pascoe, 1864. *Athylia* presently consists of two subgenera: *Athylia* (*s. str.*) with 22 species and one subspecies, and *Pulchrathylia* Breuning, 1964 with two species. All species and subspecies of the above subgenera are distributed in the Oriental Region (Tavakilian and Chevillotte 2023). Breuning (1938, 1968a) described *Mispilaassamensis* from India (Assam) and Mispila (Dryusa) coomani from Vietnam (Tonkin), respectively. Yan et al. (2023) and Xie et al. (2023) revised some species of *Mispila* Pascoe, 1864. *Mispila* currently consists of three subgenera: *Mispila* (*s. str.*) with 37 species and one subspecies, *Dryusa* Pascoe, 1864 with six species, and *Trichomispila* Breuning, 1939 with two species. All species and subspecies of the above subgenera are distributed in East, South, and Southeast Asia, as well as in Oceania (Tavakilian and Chevillotte 2023).

During the study of the genera *Souvanna*, *Athylia*, and *Mispila*, we found that the taxonomic status of *Athyliaquadristigma*, *Souvannaphoumai*, *Mispilacoomani*, and *Mispilaassamensis* were doubtful; additionally, the gender of the holotype of *Alidusmultilineatus* Pic, 1925 (currently, a junior synonym of *Mispilacurvilinea* Pascoe, 1869) was unknown. Therefore, we studied these issues and report our results here.

## ﻿Material and methods

Specimens examined are deposited in following institutions and private collections:

**BMNH**The Natural History Museum, London, United Kingdom;

**BPBM**Bernice Pauahi Bishop Museum, Honolulu, Hawaii, USA;

**CAS**California Academy of Sciences, San Francisco, CA, USA;

**CSG** Collection Andre Skale, Gera, Germany;

**CWW** Collection Andreas Weigel, Wernburg, Germany;

**LPSNU** School of Biological Science and Technology, Liupanshui Normal University, Liupanshui, Guizhou, China;

**MNHN**Muséum national d’Histoire naturelle, Paris, France;

**SYSU**Sun Yat-sen University, Guangzhou, Guangdong, China.

**Figure 1.**
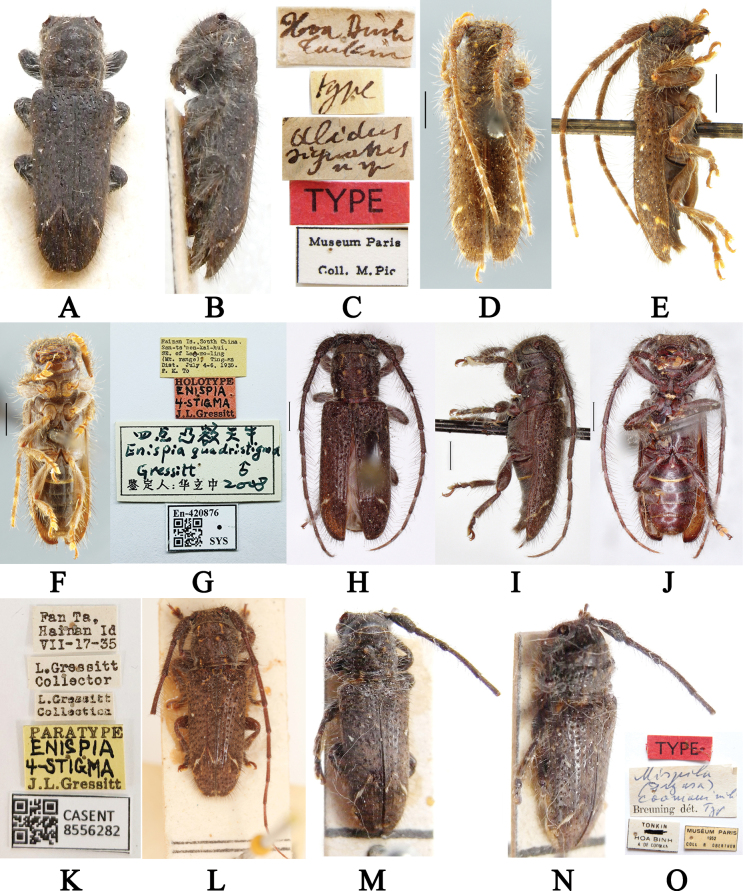
**A–C***Alidussignatus*, lectotype **A** dorsal habitus **B** lateral habitus **C** labels **D–K***Enispiaquadristigma***D–G** holotype **D** male dorsal habitus **E** male lateral habitus **F** male ventral habitus **G** labels **H–K** paratype **H** male dorsal habitus **I** male lateral habitus **J** male ventral habitus **K** labels **L***Souvannaphoumai*, syntype, dorsal habitus **M–O***Mispilacoomani*, holotype **M** dorsal view **N** lateral view **O** labels. Scale bars: 1 mm (**D–F, H–J**).

**Figure 2.**
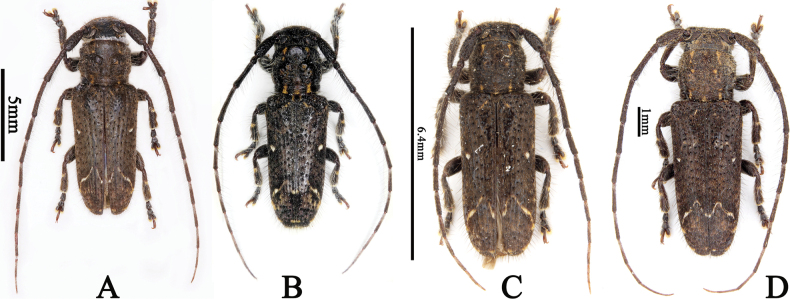
*Souvannasignata*, dorsal habitus **A** male from Yunnan, China **B** male from Trat, Thailand **C** male from Thái Nguyên, Vietnam **D** female from Thừa Thiên Huế, Vietnam.

**Figure 3.**
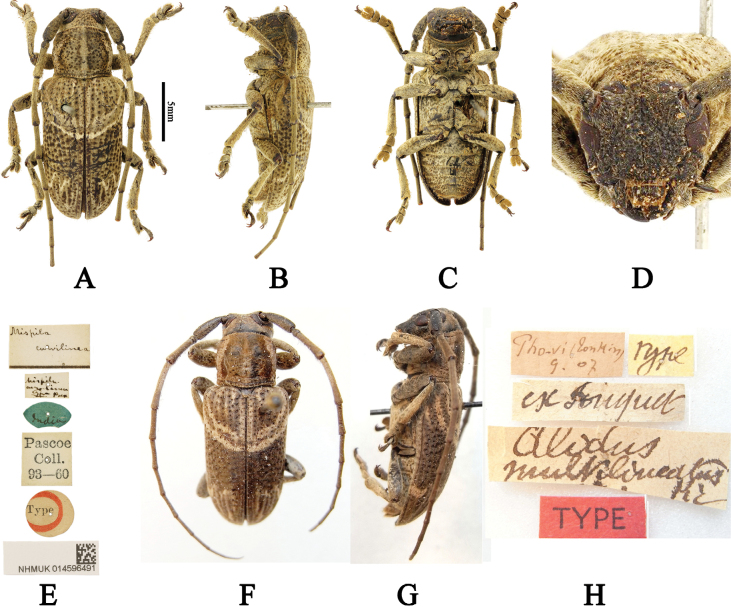
**A–E***Mispilacurvilinea*, holotype (photographs of Fig. 3A–C, E reproduced from Xie et al. 2023) **A** male dorsal habitus **B** male lateral habitus **C** male ventral habitus **D** male frontal view **E** labels **F–H***Alidusmultilineatus*, holotype **F** male dorsal habitus **G** male lateral habitus **H** labels.

**Figure 4.**
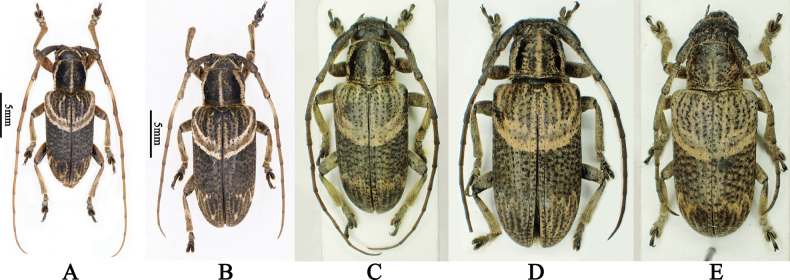
*Mispilacurvilinea*, dorsal habitus **A** male from Yunnan, China **B** female from Yunnan, China **C** male from Hua Phan, Laos **D** female from Hua Phan, Laos **E** female from Pahang, Malaysia.

**Figure 5.**
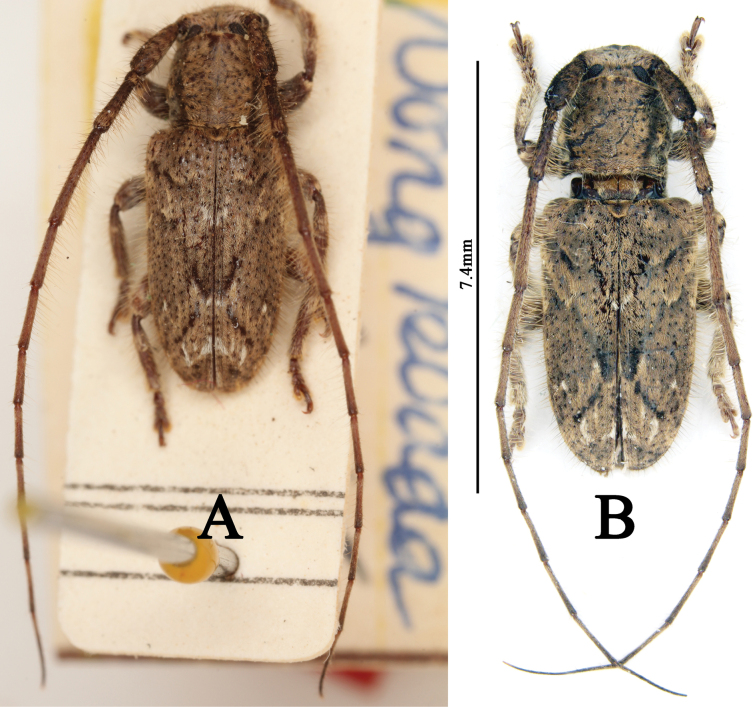
*Mispilasubtonkinea*, males, dorsal habitus **A** holotype **B** specimen from Vietnam.

**Figure 6.**
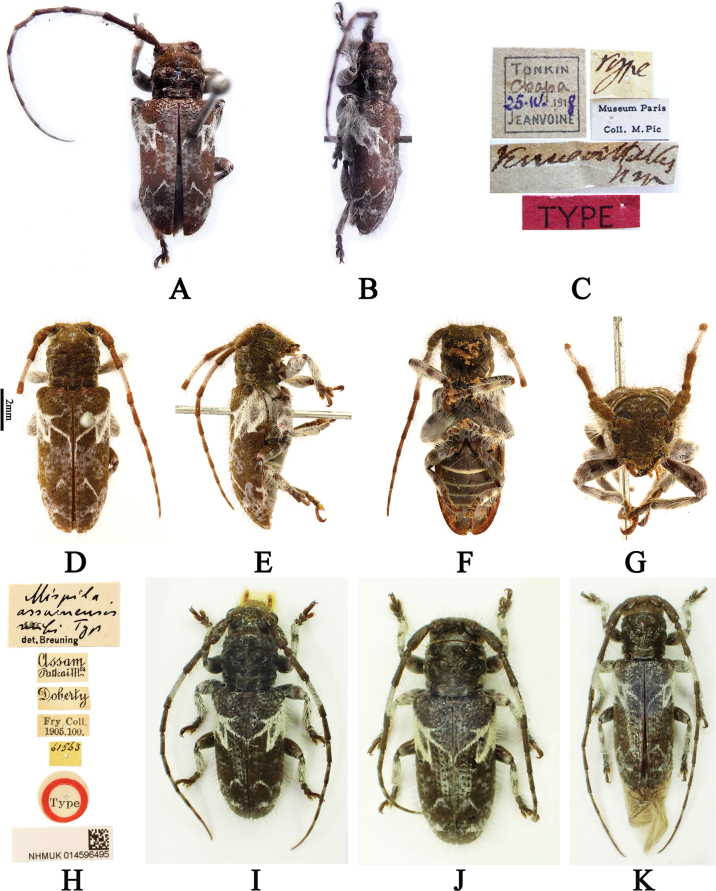
*Mispilatenuevittata***A–C***Sodustenuevittatus*, holotype **A** male dorsal habitus **B** male lateral habitus **C** labels (photographs of Fig. 6A–C reproduced from Yan et al. 2023) **D–H***Mispilaassamensis*, holotype (photographs of Fig. 6D–F, H reproduced from Xie et al. 2023) **D** female dorsal habitus **E** female lateral habitus **F** female ventral habitus **G** female frontal view **H** labels **I** male dorsal habitus, from Phang-nga, Thailand **J** female dorsal habitus, from Ranong, Thailand **K** male dorsal habitus, from Thừa Thiên Huế, Vietnam.

Photographs of Figs 1A–C, M–O, 3F–H were taken by Xavier Gouverneur, Fig. 1D–G by Bing-Lan Zhang, Fig. 1H–K by Rachel Diaz-Bastin, Figs 1L, 5A by Nobuo Ohbyashi, Figs 3D, 6G by Guang-Lin Xie, Figs 2A, 4A, B by En-Ming Chang, and Figs 2B–D, 4C–E, 5B, 6I–K by Andreas Weigel. All photographs were edited with Adobe Photoshop CS 5.

## ﻿Taxonomy

### ﻿Apomecynini

#### ﻿ Souvanna

Taxon classificationAnimaliaColeopteraCerambycidae

Breuning, 1963

228E4A6B-3F0B-5EBD-917D-530800A95499

Souvanna Breuning, 1963a: 39: Breuning 1964a: 5; Breuning 1964b: 428. Type species: Souvannaphoumai Breuning, 1963.

#### ﻿ Souvanna signata

Taxon classificationAnimaliaColeopteraCerambycidae

(Pic, 1926) comb. nov.

F431B15C-6C83-5933-9F5B-DA9195EAF7DD

Figs 1 , 2

Alidus signatus Pic, 1926: 13 (type locality “Tonkin, Vietnam”).
Mispila (Mispila) signata : Breuning 1961: 281 (catalogue); Breuning 1963b: 473 (key), 478 (redescription).
Enispia quadristigma Gressitt, 1940: 156 (type locality “Central Hainan: Sam-ts ‘uen-kai-hui, southeast of Lai-mo-leng and Fan-ta, southeast of Nam-fung”), pl. 4, fig. 12; Gressitt 1951: 482 (catalogue); Breuning 1963c: 5; Hua et al. 2009: 76, pl 76, fig. 871 (holotype, ♂). syn. nov.
Athylia quadristigma : Breuning 1960: 166 (catalogue); Hua 1982: 69 (catalogue); Hua 2002: 197 (catalogue); Löbl and Smetana 2010: 230 (catalogue); Lin and Tavakilian 2019: 331 (catalogue); Danilevsky 2020: 323 (catalogue).
Souvanna phoumai Breuning, 1963a: 39 (type locality “région de Vientiane, Laos”), figs pp. 39 and 40; Breuning 1964b: 429; Rondon and Breuning 1970: 365 (catalogue), fig. 12h. syn. nov.
Mispila (Dryusa) coomani Breuning, 1968a [nec Mispilacoomani (Pic, 1934)]: 858 (type locality “Hoa Binh, Tonkin, Vietnam”). syn. nov.

##### Body length.

6.5–10.7 mm (♂), 9.6 mm (♀). The gender of the types of *Alidussignatus* (lectotype), *Souvannaphoumai* (syntype), and *Mispilacoomani* (holotype) were unknown, so the body length was determined from the types of *Enispiaquadristigma* and additional materials examined.

##### Type material examined.

***Alidussignatus***: lectotype (MNHN), Hoa Binh Tonkin (handwritten with black ink on a rectangular white label) / type (handwritten with black ink on a rectangular white label) / *Alidussignatus* n sp (handwritten with black ink on a rectangular white label) / TYPE (printed with black ink on a rectangular red label) / Museum Paris Coll. M. Pic (printed with black ink on a rectangular white label with black borders); examined from three photographs (Fig. 1A–C). ***Enispiaquadristigma***: holotype, ♂ (SYSU), Hainan Is., South China. Sam-ts’ uen-kai-hui. SE. of Lai-mo-ling (Mt. range). Ting-an Dist. July 4–6. 1935. F. K. To (printed with black ink on a rectangular yellow label) / HOLOTYPE ENISPIA 4-STIGMA J.L. Gressitt (“HOLOTYPE J.L. Gressitt” printed and “ENISPIA 4-STIGMA” handwritten with black ink on a rectangular red label) / 四点凸额天牛*Enispiaquadristigma* Gressitt ♂ 鉴定人:华立中 2008 (“四点凸额天牛*Enispiaquadristigma* Gressitt ♂ 2008” handwritten and “鉴定人:华立中” printed with black ink on a rectangular white label with black borders) / En-420876 SYS plus a QR-code (printed with black ink on a rectangular white label with black borders); examined from four photographs (Fig. 1D–G); paratype, ♂ (CAS), Fan Ta, Hainan Id VII-17-35 (printed with black ink on a rectangular white label) / L. Gressitt Collector (printed with black ink on a rectangular white label) / L. Gressitt Collection (printed with black ink on a rectangular white label) / PARATYPE ENISPIA 4-STIGMA J.L. Gressitt (“PARATYPE J.L. Gressitt” printed and “ENISPIA 4-STIGMA” handwritten with black ink on a rectangular yellow label) / CASENT 8556282 plus a QR-code (printed with black ink on a rectangular white label with black borders); examined from four photographs (Fig. 1H–K). ***Souvannaphoumai***: syntype, ♂ (BPBM), région de Vientiane, Laos, XI.1962; examined from one photograph (Fig. 1L). **Mispila (Dryusa) coomani**: holotype (MNHN), TYPE (printed with black ink on a rectangular red label) / Mispila (Dryusa) coomani mihi Breuning dét. Typ [“Mispila (Dryusa) coomani mihi typ” handwritten with blue ink and “Breuning dét.” printed with black ink on a rectangular white label]/ TONKIN HOA BINH A DE COOMAN (printed with black ink on a rectangular white label with black borders) / MUSÉUM PARIS 1952 COLL R OBERTHUR (printed with black ink on a rectangular white label with black borders); examined from three photographs (Fig. 1M–O).

##### Additional material examined.

**China** • 1♂ (LPSNU, fig. 2A), Menglun Reservoir, Menglun Town, Mengla County, Xishuangbanna Dai Autonomous Prefecture, Yunnan Province, 5.XII.2016, leg. Ri-Xin Jiang. **Thailand** • 1♂ (CSG, fig. 2B): Ko Kut Isl., Trat Province, 24.V–8.VI. 2022, leg. A. Skale. **Vietnam** • 1♂ (CWW, fig. 2C), vic. Me Linh (IEBR station) [Institute of Ecology and Biological Resources], vic. Ngoc Thanh, Thái Nguyên Province, 21°23'3"N, 105°42'44"E, Alt. 60–80 m, 12.V.2012, leg. A. Weigel • 1♀ (CWW, fig. 2D): River valley, Son O Lau, ca. 30 km NW Hue, Thừa Thiên Huế Province, 16°31'3"N, 107°15'36"E, Alt. 30 m, 11.V.2019, leg. A. Weigel, KL [umbrella].

##### Comments.

Having compared the types of *M.signata* (Fig. 1A, B), *A.quadristigma* (Fig. 1D–F, H–J), *S.phoumai* (Fig. 1L[REMOVED HYPERLINK FIELD]) and *M.coomani* (Fig. 1M, N), we found that the above four species are identical; thus, we treat *A.quadristigma*, *S.phoumai* and *M.coomani* as junior synonyms of *M.signata*.

*Athylia* and *Souvanna* belong to the tribe Apomecynini, while *Mispila* belongs to the tribe Pteropliini. According to the key to tribes of Lamiinae and the key to genera of Apomecynini (Rondon and Breuning 1970), the difference between Apomecynini and Pteropliini is that the middle tibiae have an outer groove in front of the apex in Apomecynini and the middle tibiae have no outer groove in front of the apex in Pteropliini. The difference between *Athylia* and *Souvanna* is that the eyes are deeply emarginated in *Souvanna* and subdivided in *Athylia*. Furthermore, according to Breuning (1964b) and our examined materials, the 3^rd^ antennal joint is longer than the 4^th^ in *Athylia*, while the 3^rd^ antennal joint is shorter than the 4^th^ in *Souvanna*. To confirm these differences, we also have examined material of *Athyliaavara* Pascoe, 1864 from the Ternate Island (Indonesia), the type species of *Athylia*.

After our examination of these materials, we can confirm that this species belongs to the tribe Apomecynini, and, according to the mentioned features, it belongs to the monotypical genus *Souvanna*.

In the description of *Alidussignatus*, Pic (1926) mentioned “Long. 7–9 mill”, which indicates that Pic examined at least two specimens; thus, we designate syntype examined by us (Fig. 1A, B) as the lectotype following the recommendations of ICZN (1999; Art. 74.7).

##### Distribution.

China (Hainan, Yunnan), Laos (Vientiane), Thailand (Trat), Vietnam (Hoa-Binh, Thái Nguyên, Thừa Thiên Huế).

### ﻿Pteropliini

#### ﻿ Mispila

Taxon classificationAnimaliaColeopteraCerambycidae

Pascoe, 1864

2C28DF36-4C35-5979-ADD7-59DBF4A66E44

Mispila Pascoe, 1864: 90: Gemminger and Harold 1873: 3092; Hüdepohl 1995: 295; Kariyanna et al. 2017: 192. Type species: Mispilavenosa Pascoe, 1864.
Mispila (Mispila) Aurivillius 1922: 275; Breuning 1961: 281; Breuning 1963b: 471 (redescription); Rondon and Breuning 1970: 380 (key), 414 (key).
Diatylus Lacordaire 1872: 565. Type species: Diatyluszonarius Lacordaire, 1872.

#### ﻿ Mispila (s. str.) curvilinea

Taxon classificationAnimaliaColeopteraCerambycidae

Pascoe, 1869

A5DA5DA4-738F-513B-8942-6B0CA91F3101

Figs 3 , 4

Mispila curvilinea Pascoe, 1869: 206 (type locality “India”); Gemminger and Harold 1873: 3092 (catalogue); Hua 1982: 96 (catalogue); Hua et al. 2009: 91, pl. 91, fig. 1051 (♂, ♀); Weigel 2012: 411 (distribution), pl. 32, fig. h; Kariyanna et al. 2017: 193 (catalogue); Barševskis 2018: 291 (distribution).
Mispila (Mispila) curvilinea Aurivillius, 1922: 275 (catalogue): Breuning 1961: 281 (catalogue); Breuning 1963b: 472 (key), 474 (redescription); Rondon and Breuning 1970: 415 (catalogue), fig. 23 i (♀); Hua 1981: 178 (new distribution); Hua 2002: 216 (catalogue); Löbl and Smetana 2010: 315 (catalogue); Lin and Tavakilian 2019: 366 (catalogue); Danilevsky 2020: 451 (catalogue); Xie et al. 2023: 248 (catalogue), fig. 3 (a–d holotype, ♂; e, f ♀; g, h ♂).
Alidus multilineatus Pic, 1925: 24 (type locality “Tonkin, Vietnam”): Breuning 1963b: 475 (synonymized).

##### Body length.

15.0–18.0 mm (♂), 12.0–18.2 mm (♀). The body length was determined from the holotypes of *Mispilacurvilinea*, *Alidusmultilineatus*, and additional materials examined. The body length of the holotype of *A.multilineatus* was mentioned in the original paper (Pic 1925).

##### Type material examined.

***Mispilacurvilinea***: holotype, ♂ (BMNH), *Mispilacurvilinea* (handwritten with black ink on a rectangular white label with a straight-line black border) / *Mispilacurvilinea* typ Pasc (handwritten with black ink on a rectangular white label) / India (handwritten with black ink on a fan-shaped green label) / Pascoe Coll. 93–60 (printed with black ink on a square white label) / Type (printed with black ink on a circular white label with circular red borders) / NHMUK 014596491 plus a QR-code (printed with black ink on a rectangular white label); examined from five photographs (Fig. 3A–E). ***Alidusmultilineatus***: holotype, ♂ (MNHN), Pho-vi (Tonkin) 9. 07 (handwritten with black ink on a rectangular white label) / Type (handwritten with black ink on a rectangular yellow label) / ex Buquet (handwritten with black ink on a rectangular white label) / *Alidusmultilineatus* Pic (handwritten with black ink on a rectangular white label) / TYPE (printed with black ink on a rectangular red label); examined from three photographs (Fig. 3F–H).

##### Additional material examined.

**China** • 3♂♂, 3♀♀ (LPSNU), Hulukou, Xima Town, Yingjiang County, Dehong Dai and Jingpo Autonomous Prefecture, Yunnan Province, Alt. 1200 m, VI–VII.2018, leg. Wei-Zong Yang • 1♂ (CWW): vic. Guo Men Shan (NNNR), 37 km NW Jinghong City, Xishuangbanna Dai Autonomous Prefecture, Yunnan Province, 22°14'43"N, 100°36'12"E, Alt. 1100 m, 18.VI.2019, leg. A. Weigel LFF [light trap]. **Laos**: 1♂, 2♀♀ (CWW): Phou Pan (Mt.), Ban Saleui, Hua Phan Province, 20°12'N, 104°01'E, Alt. 2060 m, V.2017, leg. local collector. **Malaysia** • 1♀ (CWW, fig. 4E): 35 km SE Ipoh, Tanah Rata, Cameron Highland, Pahang, 4°28'N, 101°23'E, Alt. 1500 m, 19–31.III.2003, leg. M. Nèmec.

##### Comments.

In the original description of *A.multilineatus*, Pic (1925) did not mention the gender of the holotype. Breuning (1963b: 475) treated it as a junior synonym of *M.curvilinea* and provided a supplementary description of this species, including the sexual differences: “antennae are more than 0.5 time longer than body in males, antennae are slightly shorter than body in females.” According to above characters and the material examined, we could confirm that the holotype of *A.multilineatus* is a male.

##### Distribution.

Bengal (Klimpong, Samsingh), Cambodia, China (Guangxi, Yunnan), India (Sikkim), Laos (Bokeo, Hua Phan, Khammouane, Vientiane), Malaysia (Pahang), Vietnam (Ha Giang).

#### ﻿ Mispila (s. str.) subtonkinea

Taxon classificationAnimaliaColeopteraCerambycidae

Breuning, 1968

850A1848-0077-5D83-A566-6C81A9AC7A0C

Fig. 5

Mispila (*s. s.*) subtonkinea Breuning, 1968b: 21 (type locality “Vientiane, Laos”).
Mispila (*s. str.*) subtonkineaRondon and Breuning 1970: 415 (catalogue), fig. 24a (holotype, ♂); Xie et al. 2023: 260 (catalogue), fig. 13g–l (♂).

##### Body length.

8.0–9.0 mm (♂). The body length is determined from the holotype of *M.subtonkinea* and additional materials examined. The body length of the holotype of *M.subtonkinea* is referred to in the original paper (Breuning 1968b).

##### Type material examined.

***Holotype***, ♂ (BPBM), Vientiane, Laos, 15 février (= February) 1965; examined from one photograph (Fig. 5A).

##### Additional material examined.

**Vietnam**: 1 ♂ (CWW, fig. 5B): 6 km SW von Thanh Son, Tay Yen Tu Nat. Res., Bắc Giang Province, 21°10.830'N, 106°43.427'E, Alt. 200 m, 18–21.V.2015, leg. A. Weigel, KS [clearing] LFF [light trap].

##### Distribution.

Laos (Vientiane), Vietnam (Bắc Giang).

#### ﻿ Mispila (s. str.) tenuevittata

Taxon classificationAnimaliaColeopteraCerambycidae

(Pic, 1930)

EE2EF8DC-B90E-5F6D-B9CC-0AC19255E48A

Fig. 6

Sodus tenuevittatus Pic, 1930: 19 (type locality: “Chapa, Tonkin, Vietnam”).
Mispila (Mispila) venosa m. tenuevittata: Breuning 1961: 281 (catalogue); Breuning 1963b: 486 (catalogue).
Mispila (*s. str.*) tenuevittata: Yan et al. 2023: 4 (stat. resurrected, catalogue), figs 2A–H, 3A–D; Xie et al. 2023: 255 (catalogue), fig. 8 (e–g ♂, h–j ♀).
Mispila (Dryusa) sonthianae Breuning, 1963d: 59 (type locality: “Vientiane, Laos”), fig. (holotype); Rondon and Breuning 1970: 415 (catalogue), fig. 24b (holotype, ♂); Löbl and Smetana 2010: 315 (catalogue); Lin and Tavakilian 2019: 366 (catalogue); Danilevsky 2020: 451 (catalogue).
Mispila sonthianae : Hua 2002: 216 (catalogue); Hua et al. 2009: 92, pl. 92, fig. 1053 (♂, ♀).
Mispila assamensis Breuning, 1938: 381 (type locality: “Mtes. Patkai, Assam, India”); Kariyanna et al. 2017: 193 (catalogue); Mitra et al. 2017: 87 (distribution); Xie et al. 2023: 257 (catalogue), fig. 11 (a–d holotype, ♀; e–h ♂). syn. nov.
Mispila (Mispila) assamensis : Breuning 1961: 281 (catalogue); Breuning 1963b: 472 (key), 487 (redescription).

##### Body length.

7.1–12.0 mm (♂), 7.6–13.7 mm (♀). The body length was determined from Yan et al. (2023), the holotype of *M.assamensis*, and additional materials examined.

##### Type material examined.

***Sodustenuevittatus***: see Yan et al. 2023. ***Mispilaassamensis***: holotype, ♀ (BMNH), *Mispilaassamensis* mihi Typ det. Breuning (“*Mispilaassamensis* mihi Typ” handwritten and “det. Breuning” printed with black ink on a rectangular white label) / Assam Patkai Mt. (handwritten with black ink on a rectangular white label) / Doherty (handwritten with black ink on a rectangular white label)/ Fry Coll. 1905. 100. (printed with black ink on a rectangular white label) / 61563 (handwritten with black ink on a rectangular white label) / Type (printed with black ink on a circular white label with circular red borders) / NHMUK 014596495 plus a QR-code (printed with black ink on a rectangular white label); examined from five photographs (Fig. 6D–H).

##### Additional material examined.

**Thailand** • 1♂ (CWW): vic. Khao Lak, Takuapa distr., Phang-nga Province, 08°37.623'N, 98°15.091'E, Alt. 50 m, 23.VIII–02.IX.2010, leg. A. Skale • 3♂♂, 3♀♀ (CWW): vic. Khao Lak, Takuapa distr., Phang-nga Province, 08°37'N, 98°15'E, 07.VIII.2012, leg. A. Weigel • 1♀ (CWW, fig. 6J): Yai island, Kam, 14 km W Na Kha, Ranong Province, 09°29.652'N, 98°21.385'E, 09.VIII.2012, leg. A. Weigel, UWP [primary forest] KÜ [coast]. **Vietnam** • 2♂♂ (CWW, fig. 6K): Son Hurang River, Hue, Thừa Thiên Huế Province, 16°27'36"N, 107°34'7"E, 10 m [a. s. l.], 11.V.2019, leg. A. Weigel, LFF [light trap] (Hotel).

##### Comments.

After having compared the holotypes of *Mispilatenuevittata* (Fig. 6A, B) and *M.assamensis* (Fig. 6D–G), we found that both holotypes are identical, except for the different genders. Thus, we treat *M.assamensis* as a junior synonym of *M.tenuevittata*. Yan et al. (2023) marked the holotype of *M.tenuevittata* as a female in the legend, while they considered it as a male in the comments.

##### Distribution.

China (Guangxi, Hainan, Yunnan), India (Assam), Laos (Bokeo, Mekong, Vientiane), Thailand (Phang-nga, Ranong), Vietnam (Chapa, Thừa Thiên Huế).

## Supplementary Material

XML Treatment for Souvanna

XML Treatment for Souvanna signata

XML Treatment for Mispila

XML Treatment for Mispila (s. str.) curvilinea

XML Treatment for Mispila (s. str.) subtonkinea

XML Treatment for Mispila (s. str.) tenuevittata

## ﻿Additional information

### Author ORCIDs

Gui-Qiang Huang https://orcid.org/0000-0002-0063-8157

Andreas Weigel https://orcid.org/0000-0002-3237-6614

En-Ming Chang https://orcid.org/0009-0000-2039-1318

Gui-Mei Zhang https://orcid.org/0000-0002-4969-3288

### Data availability

All of the data that support the findings of this study are available in the main text.
